# Moments of genome evolution by Double Cut-and-Join

**DOI:** 10.1186/1471-2105-16-S14-S7

**Published:** 2015-10-02

**Authors:** Priscila Biller, Laurent Guéguen, Eric Tannier

**Affiliations:** 1 Institute of Computing, University of Campinas, São Paulo, Brazil; 2 Institut National de Recherche en Informatique et en Automatique (INRIA) Grenoble Rhône-Alps, 655 avenue de L'Europe, 38330 Montbonnot, France; 3 Laboratoire de Biométrie et Biologie Évolutive, LBBE, UMR CNRS 5558, University of Lyon 1, 43 boulevard du 11 novembre 1918, 69622, Villeurbanne, France

**Keywords:** coagulation-fragmentation, inversion, rearrangement, method of moments, random graphs, statistical inference

## Abstract

We study statistical estimators of the number of genomic events separating two genomes under a Double Cut-and Join (DCJ) rearrangement model, by a method of moment estimation. We first propose an exact, closed, analytically invertible formula for the expected number of breakpoints after a given number of DCJs. This improves over the heuristic, recursive and computationally slower previously proposed one. Then we explore the analogies of genome evolution by DCJ with evolution of binary sequences under substitutions, permutations under transpositions, and random graphs. Each of these are presented in the literature with intuitive justifications, and are used to import results from better known fields. We formalize the relations by proving a correspondence between moments in sequence and genome evolution, provided substitutions appear four by four in the corresponding model. Eventually we prove a bounded error on two estimators of the number of cycles in the breakpoint graph after a given number of rearrangements, by an analogy with cycles in permutations and components in random graphs.

## Introduction

Double Cut and Join (DCJ) is a mathematical operator modeling genome rearrangements which has considerably simplified many combinatorial studies [1] compared with other operators. We would like to show here how it can also significantly enrich and simplify statistical methods of moment estimations. These consist in computing an expected value for some parameter *p *after a fixed number *k *of DCJ applied to a genome. The parameter can be the number of breakpoints (gene neighborhood present in the initial genome but not in the final one), or the number of cycles in the breakpoint graph (a slightly more complicated structure defined later). Then, by the method of moments, an estimate of *k*, which is usually unknown, can be computed as a function of *p*, which has an observed value, by inverting the expected value of *p *as a function of *k*.

There have been a few published probabilistic models for DCJ, usually giving equal probability to every event coded as a DCJ. They lead to heuristic estimators of the number of breakpoints between two genomes after a fixed number of DCJs [2,3], Bayesian sampling strategies among evolutionary scenarios [4], estimates of the domain of validity of parsimony [5], or estimates of transposition rates [6].

Statistical methods related to inversions (see among others [7-10]) show a variety of techniques and build informal links with various known processes as random graphs, transpositions in the symmetric group, and coagulation-fragmentation. This allows one to adapt statistical results from other fields to genome rearrangements. Another way to do so is to code genome arrangements by sequences or binary characters and let these sequences evolve by substitutions [11,12]. The efficiency of these importations has empirically been tested on simulations, but has not been assessed theoretically.

Here we introduce a "mechanistic" DCJ model, based on breakage probabilities rather than on events, which allows one to

**Figure 1:**
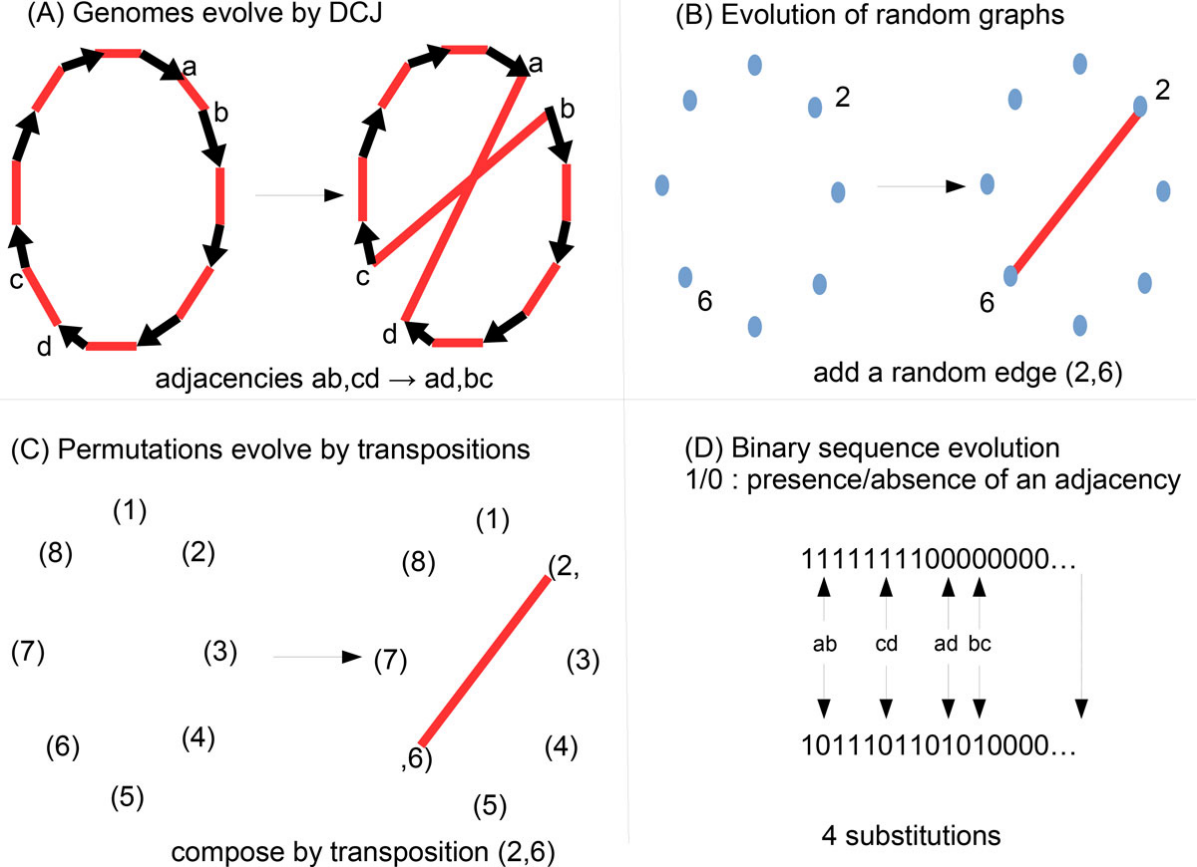
**Evolution of genomes, sequences, permutations or graphs**. Similarity between four processes: (A) counting the number of breakpoint or cycles in the breakpoint graph of two genomes evolved by DCJ; (B) counting the number of components in a random graph; (C) counting the number of cycles in a permutation evolved from the identity by transpositions; (D) counting the number of different sites in two binary sequences evolved by substitutions.

• Obtain a closed, analytically invertible, exact formula for the expected number of breakpoints after a fixed number of DCJs; the previously published estimation [2] was based on an unbounded approximation, computed by a recurrence and thus not easily invertible.

• Establish formal links with three well-known processes, and in consequence theoretically found or correct the intuitions of former studies. A graphical intuition of these links is drawn in Figure 1. We show that coding genome arrangements by binary structures gives estimations only if substitutions are supposed to occur four by four, which has the same effect as adjusting the size of the sequence. Without this correction the estimations are badly wrong as shown from simulations. Then we show that the random graphs or transpositions in the symmetric group induce an estimation error less than $O\left(k/\sqrt{n}\right)$ if used for DCJ, where *n *is the number of genes. As saturation occurs at *k *= *O*(*n *log *n*), the error is always bounded by *o*(*n*). In practice, on simulations, it does not make a visible difference.

We first describe our model for evolving genomes by DCJ and some of its properties.

## Genomes and DCJ

Here a *genome *is defined as a graph on a set of 2*g *vertices, called *gene extremities*, composed by two matchings. Recall a *matching *is a set of edges (unoriented pairs of vertices) or arcs (oriented pairs of vertices) such that any two edges (or arcs) in the set do not share vertices. In a genome one matching has *g *arcs, called *genes*, and the other has *a *≤ *g *edges, called *adjacencies*. The 2*g *− 2*a *gene extremities that do not belong to an adjacency are called *telomeres*. This definition models gene order in linear or circular chromosomes: genes as arcs model oriented segments of DNA, and adjacencies are the links between consecutive genes on a chromosome, being more general than *signed permutations *[1].

**Figure 2:**
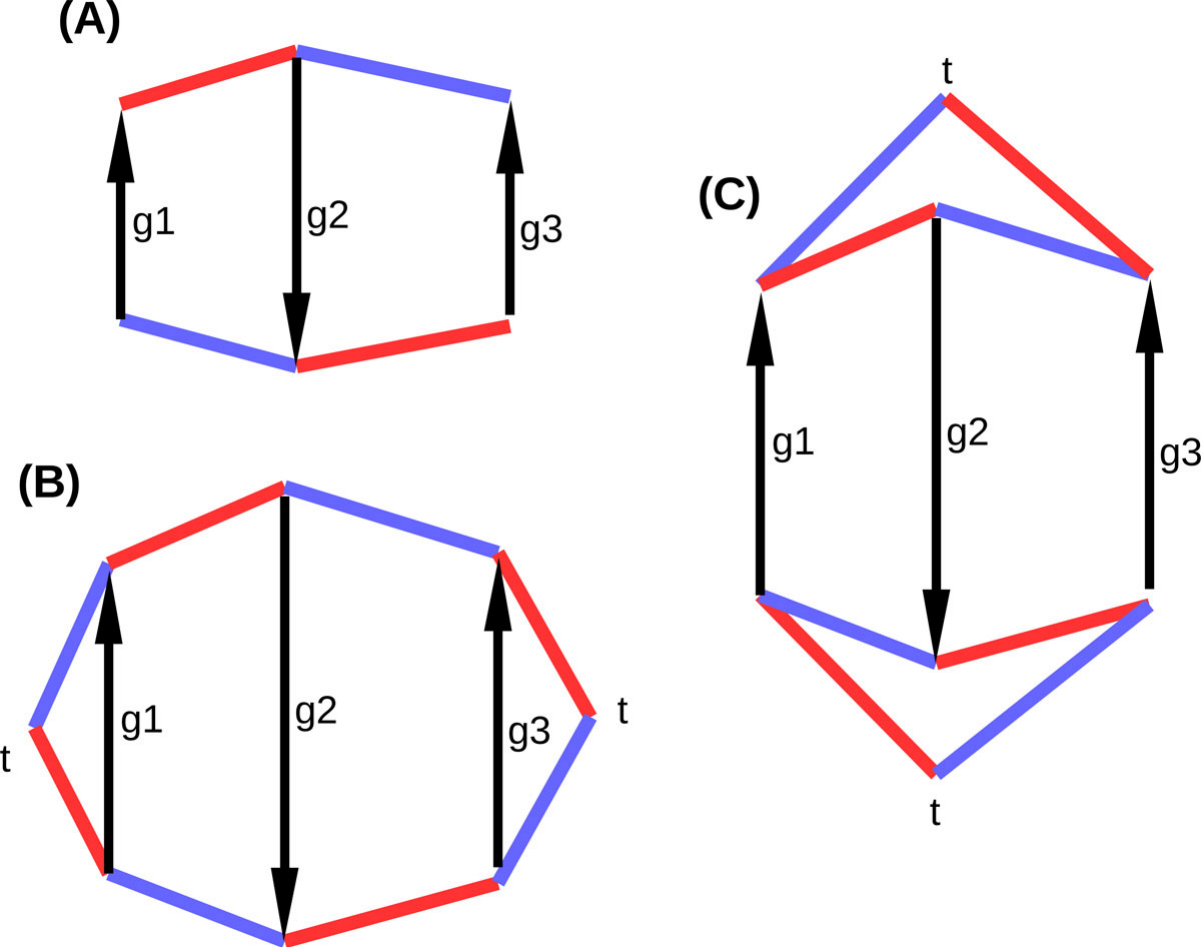
**Two genomes, with their real and observed breakpoint graphs**. Genes are *g*1, *g*2, *g*3, ordered in one linear chromosome in a blue and a red genome: in the order *g*1*g*2*g*3 on the red chromosome, and in the order *g*3*g*2*g*1 on the blue chromosome. In (A) the two non augmented genomes are depicted. In (B) and (C), augmented genomes are depicted. Both augmented genomes are identical in both parts. Only the correspondence of the telomeric vertices between the blue and red genomes vary, which illustrates the possible difference between the real and observed breakpoint graph. There are two breakpoints in the red genome with respect to the blue genome. In the situation (B) the blue genome can be obtained from the red by three DCJs. But the adjacencies leading to *t *vertices are not observed, so another possibility is (C), where a scenario with only two DCJs exist. Case (C) is the observed breakpoint graph because it maximizes the number of cycles in the breakpoint graph. The real breakpoint graph can be either (B) or (C), but to determine it requires a knowledge of the evolutionary scenario that have shaped these two genomes.

When we compare two genomes, we assume that they are on the same set of vertices, and that the genes are the same. Only adjacencies are different. So the arcs are used only to make the connection between matchings and gene orders, but can be ignored in the comparisons. For example, Figure 2(A) shows two genomes (the red matching and the blue matching) with three genes, six gene extremities, and two adjacencies. The red matching yields gene order *g*1*g*2*g*3 and the blue matching *g*3*g*2*g*1.

For technical purposes we define an *augmented genome *by adding a so-called *telomeric vertex t *for each telomere *x*, and a *telomeric adjacency *between *x *and *t*. We also introduce an even number *f *(*f *will be a parameter of the model) of *fictional vertices *that are perfectly matched two by two by *fictional adjacencies *in an arbitrary way. No vertex remains unmatched in the augmented genome, which has 4*g *− 2*a *+ *f *vertices and *n *= 2*g *− *a *+ *f*/2 edges. We call the non telomeric and non fictional vertices (or adjacencies) as *observed*.

For example, an augmented genome for the red and the blue genomes in Figure 2 (A)is depicted in Figure 2(B) or 2(C). There are two observed and two telomeric adjacencies in the red and blue genomes, two telomeric and no fictional vertices. Note that we still require that the telomeric vertices, as all gene extremities, are shared between the two compared genomes, but there are several different ways to do so, as exemplified by Figure 2(B) and 2(C). This is discussed in the "Breakpoint graphs" section, when this distinction becomes important.

We always suppose that *f *= *o*(*n*) and 2*g *− 2*a *= *o*(*n*), that is, most gene extremities have observed adjacencies.

A Double Cut-and-Join (DCJ) is an operation defined on an augmented genome, in which two different adjacencies (of any kind) *AB *and *CD *are replaced by new adjacencies *AC *and *BD*, or *AD *and *BC*. For instance, two DCJs transform the red genome into the blue genome in Figure 2(C): one DCJ transforms the red edges of the lower cycle into the blue edges, and the same thing for the upper cycle.

This definition of DCJ contains all types of operations usually defined as DCJ on non-augmented genomes [13], including fusions, fissions, and operations including telomeres. It also contains some operations that do not affect the non augmented genome. So our definition is equivalent to the usual one if we add an "do nothing" operation.

A DCJ can change a telomeric vertex into fictional and *vice versa*. So the nature of telomeric and fictional vertices or adjacencies is the same regarding the evolutionary process.

The *DCJ model *of genome evolution is a Markov chain on the set of perfect matchings on graphs with 2*n *vertices, which consists in selecting two different adjacencies uniformly at random, and choosing one of the two ways to draw two adjacencies different from the initial configuration on the same four vertices.

The model is slightly different from two previously published ones [2,4]. They assumed that all operations modified the non augmented genome and that all distinct such operations were equiprobable. As some operations in our definition do nothing to the non augmented genome, and some distinct operations on the augmented genome have the same effect on the non augmented one, some probabilities are slightly different. But the probability of the do nothing operation, provided *f *stays low compared to *n*, is low enough so that in practice it can be negligible.

Note that, with this model, there are exactly *n*(*n *− 1) different equiprobable DCJs at any time on the augmented genome. It is slightly different than defining equiprobability of all DCJs on the non augmented genomes as in previous models. In particular, in the later, the Markov chain converges to a steady state which necessarily has a number of telomeres of the order of the square root of the number of adjacencies [2]. The steady state can be far from all data on which the model is used, for example, if the number of chromosomes should be more or less stable, and less than the square root of the number of adjacencies. Adding a parameter *f *and uniform probability on the DCJs on the augmented genome is a way to allow a different steady state. The estimation of the parameter is discussed in the sequel.

## Closed formula for the expected number of breakpoints

We give here an exact, closed, easily invertible formula for the expected number of breakpoints after *k *DCJs. A *breakpoint *of a genome *G *with respect to another genome *G_k _*is an observed adjacency *AB *in *G *such that *A *and *B *are not adjacent in *G_k _*. For instance, in Figure 2 the red genome has two breakpoints with respect to the blue (and this depends only on observable data).

**Theorem 1 ***The expected number B_k _of breakpoints between G and G_k _, if G_k _is produced from G by k DCJs, is*

(1) $$E\left(B_{k}\right)=a\frac{2n-2}{2n-1}\left(1-{\left(1-\frac{1}{n-1}-\frac{1}{n}\right)}^{k}\right),$$

*where a is the number of observed adjacencies and n is the total number of (observed, telomeric, fictional) adjacencies*.

This theorem improves on the only previous estimation for DCJ [2] which was an approximate recursive computation. The exact formula in the present theorem requires the knowledge of *n*, and thus of the parameter *f *, which is part of the model. It is always possible to set up the parameter to stick to the equilibrium properties of the model of [2], thus providing a formula for an equivalent model. The proof is similar to classical corrections of sequence evolutionary models, used also in rearrangements for unsigned inversions [7].

*Proof *The idea is first to define the probability *P_xy,k _*of a couple *xy *of gene extremities which are linked by an adjacency in *G*, to be unlinked in *G_k_*. Then we have

$$E\left(B_{k}\right)=\underset{xy\;\text{observed adjacency}}{\sum }P_{xy,k}=aP_{k},$$

where *P_k _*is the *P_xy,k _*for any *xy *because the probability *P_xy,k _*does not depend on *x *and *y*.

*P_k _*can be computed from *P*_*k*−1 _by

(2) $$P_{k}=P_{k-1}q_{u}+\left(1-P_{k-1}\right)p_{s}=P_{k-1}\left(q_{u}-p_{s}\right)+p_{s},$$

where *p_s _*is the probability to cut an adjacency by one random DCJ from the model, and *q_u _*is the probability not to form an adjacency when it is absent.

It is possible to solve the recurrence in order to obtain a closed formula depending on *p_s _*and *q_u_*. As *P*_0 _= 0,

(3) $$P_{k}=\overset{k-1}{\underset{i=0}{\sum }}p_{s}{\left(q_{u}-p_{s}\right)}^{i}=p_{s}\frac{{\left(q_{u}-p_{s}\right)}^{k}-1}{q_{u}-p_{s}-1}.$$

We can easily compute *q*_u_ and *p*_u_ from the model: $p_{s}=\frac{2\left(n-1\right)}{n\left(n-1\right)}=\frac{2}{n}$ and $q_{u}=1-\frac{1}{n\left(n-1\right)}$. Plugging these into Equation (3) gives Equation (1).

Inverting the expression of *E*(*B_k_*) gives an estimator of *k *as a function of an observed value of *B_k _*:

$$\tilde{DCJ}\left(G_{1},G_{2}\right)=\frac{\text{log}\left(1-\frac{B\left(2n-1\right)}{a\left(2n-2\right)}\right)}{\text{log}\left(1-\frac{1}{n-1}-\frac{1}{n}\right)},$$

where *B *is the observed number of breakpoints between of *G*_1 _with respect to *G*_2_, and *a *is the number of observed adjacencies in *G*_1_. This estimator requires the estimation of a parameter *f *to compute *n*, which has to be common to *G*_1 _and *G*_2_. We do not have enough observations to estimate it in a statistically grounded way, but it can be chosen between 2|*a *− *a*_2_|, where *a*_2 _is the number of observed adjacencies of *G*_2_, and $\sqrt{g\;}$, where *g *is the number of genes. The lower bound is necessary to be able to transform *G*_1 _into *G*_2_, because in a DCJ the number of telomeric plus fictional vertices never vary. So fictional elements adjust the number of telomeres. The upper bound sticks to the previously published models, where at equilibrium state the number of telomeres is $O\left(\sqrt{g}\right)$.

## A link with sequence evolution

The same reasoning as in the previous section can be applied to several models of genome evolution. Caprara and Lancia [7] applied it on a model of evolution of unsigned permutations, and it is commonly used for computing distances from evolutionary models on sequences.

In our case, let for example *S *be a sequence of size *N *over the binary alphabet {0, 1}. Let the evolutionary model on the state space of sequence be a Markov chain on all possible such sequences, with equiprobable substitutions at any site (a Jukes-Cantor-like model on a binary alphabet). If at one site there is a 1, the substitution turns it into 0, and conversely. Let *S_k_s __*be a sequence obtained after *k_s _*steps of this process. We can compute the expected number *D_k_s __*of sites that have a different value in *S *and *S_k_s __*by the formula

$$E\left(D_{k_{s}}\right)=N\;P_{k_{s}},$$

where $P_{k_{s}}$ is the probability that one site is different in $S_{k_{s}}$, and it can be computed with the recurrence (see Equations (2) and (3)):

$$P_{k_{s}}=P_{k_{s}-1}\left(q_{u}-p_{s}\right)+p_{s}=p_{s}\frac{{\left(q_{u}-p_{s}\right)}^{k_{s}}-1}{q_{u}-q_{s}-1},$$

where *p_s _*= 1/*N *is the probability to change a site given that it is the same in *S *and $S_{k_{s}}$, and *q_u _*= 1 − 1/*N *is the probability not to change back a site when it is different. This gives

(4) $$E\left(D_{k_{s}}\right)\;=\frac{N}{2}\left(1-{\left(1-\frac{2}{N}\right)}^{k_{s}}\right).$$

Let us code genomes *G *and *G_k _*evolved by *k *DCJs from *G *by two aligned binary sequences *S*_1 _and *S*_2_, where each site corresponds to a possible adjacency, with a 1 in one sequence if the adjacency is present in the associated genome, and a 0 otherwise, like for example in [11,12]. A choice has to be made for the adjacencies that are neither present in *G *nor in *G_k _*. Usually they are ignored because they represent a large set of invariable sites. We show that they are very important for statistical estimation, but should not be all present.

**Proposition ***Equation (4) is equal to the expected number of breakpoints between G and G_k _if*

$$N=\;4a\frac{2n-2}{2n-1}\approx 4a,$$

and

$$k_{s}=k\frac{\text{log}\left(1-1/n-1/\left(n-1\right)\right)}{\text{log}\left(1-\left(2n-1\right)/4a\left(n-1\right)\right)}\approx 4ka/n.$$

*Proof *The number of differences *D_k _*is twice the number of breakpoints: it counts the breakpoints from one genome and from the other. So dividing the right term of Equation 4 by two and identifying the terms $\frac{N}{4}$ in Equation 4 and $a\frac{2n-2}{2n-1}$in Equation 1 gives $N=\text{4}a\frac{2n-2}{2n-1}$. Now let ${\left(1-\frac{2}{N}\right)}^{k_{s}}$be equal to ${\left(1-\frac{1}{n-1}-\frac{1}{n}\right)}^{k}$, that is, $k_{s}\text{log}\left(1-\frac{2}{N}\right)\approx 2k_{s}/N$ with $k\text{ log}\left(1-\frac{1}{n-1}-\frac{1}{n}\right)\approx k\text{ log}\left(1-2/n\right)\approx 2k/n$. This gives *k_s _*≈ *kN/n *which, with *N *≈ 4*a*, gives *k_s _*≈ 4*ka/n*.

**Figure 3:**
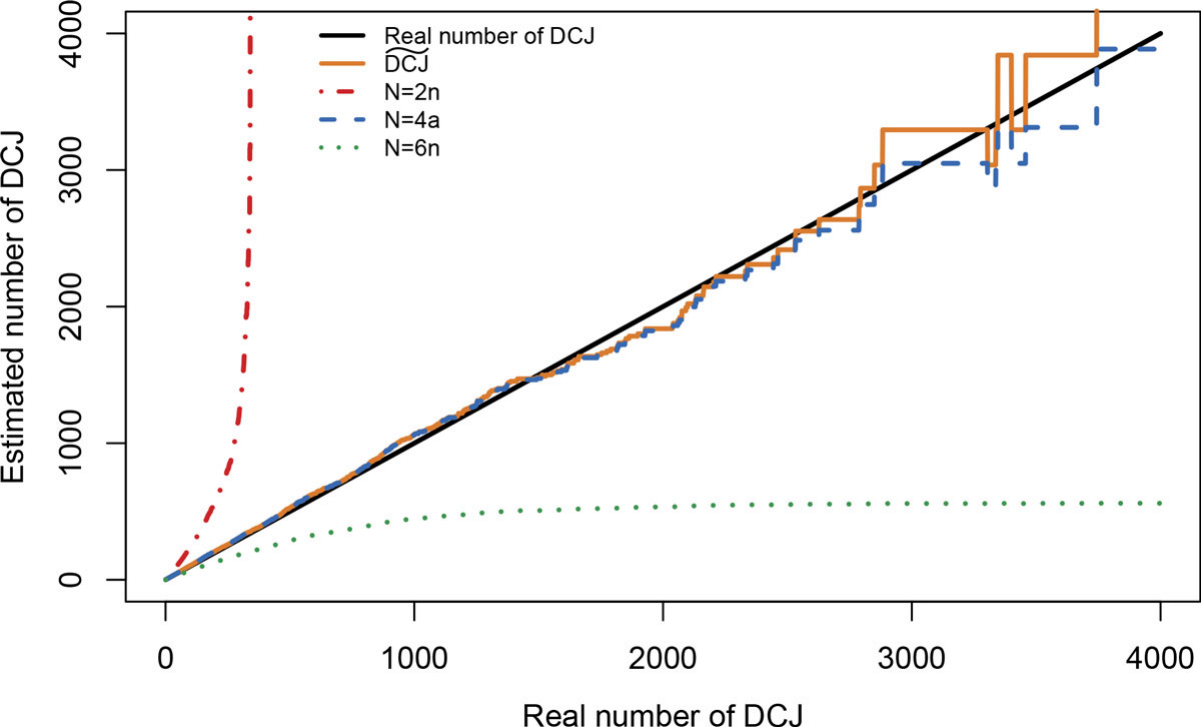
**4 × 4 correction when estimating a distance through binary sequence evolution**. A genome with *a *= 980 observed adjacencies and *n *= 1020 adjacencies in total was evolved with DCJ. The set of observed adjacencies was coded in a sequence, with a 1 for the presence of an adjacency and 0 for the absence. The number of *observable sites*, that is, the number of 0 s, was set to *N *, and variations on *N *were performed. Choosing a sequence of length *N *< 4*a*, where *a *is the number of observed adjacencies of the genomes, leads to an overestimation, while *N *> 4*a *leads to an underestimation. With *N *= 4*a*, as the theoretical results predicted, the estimation is correct and its quality is equivalent to the $\tilde{DCJ}$ direct prediction.

Simulations show that the estimation errors of codings that ignore all 0 sites, or consider them all, are not only theoretical (see Figure 3). Choosing *N *= 2*n *systematically overestimates the distance while *N *= *n*(2*n *− 1) underestimates it. With *N *= 4*a *and *k_s _*= 4*ka/n*, the estimation is quasi superposed with the DCJ estimation of Equation 1.

The intuitive reason for these multiplications by 4, and the choice of a space of possible adjacencies which is 4 times as big as the space of observed adjacencies can be understood by an adaptation of the substitution model: indeed if we think about DCJ, sequences do not evolve site per site, but as 4 simultaneous substitutions in 4 sites ("4 per 4"). A DCJ cuts two adjacencies and reforms two, which is changing 4 sites at the same time. Developing a model of sequence evolution where sites evolve 4 per 4 yields *p_s _*= 4/*N *and *q_u _*= 1 − 4/*N*, hence the expected number of differences under a 4 × 4 model is computed as follows:

$$E\left(D{\text{4}}_{k_{s}}\right)=\frac{N}{2}\left(1-{\left(1-\frac{8}{N}\right)}^{k_{s}}\right).$$

This means that a number of differences under a 4 × 4 model can be estimated from a number of differences under a single site substitution model if the length of the sequence is multiplied by 4, and the number of differences is divided by 4. This corroborates the theoretical and empirical results we obtained.

## A link with transpositions in the symmetric group

Eriksen and Hultman [9] proposed, as an analogy to signed inversions evolving by reversals, a Markov chain on the symmetric group, where permutations evolve by random transpositions. Here transpositions are permutations with one cycle of size two, or the operation of composition with these permutations. The analogy was also noted in the definition of an algebraic model of genome rearrangements [14]. Explaining the analogy requires defining breakpoint graphs.

### Breakpoint graphs

A breakpoint graph is roughly defined as the union of the adjacencies of two genomes *G*_1 _and *G*_2 _defined on the same set of genes. But this definition requires additional precision for telomeric and fictional elements because we defined them for one genome, so we need a common definition for two genomes.

There are two ways to proceed, which will end up in two different breakpoint graphs, the *real *breakpoint graph and the *observed *breakpoint graph (see Figure 2). First, suppose telomeres and fictional elements are defined on the genome *G*_1_, and *G*_2 _is evolved from *G*_1 _by a series of DCJs. Then, by extension, telomeres and fictional elements are also defined for *G*_2_, and the breakpoint graph is a set of disjoint cycles alternating between adjacencies of *G*_1 _and *G*_2_, that we call the *real breakpoint graph*.

But the real breakpoint graph cannot be observed in reality, because it would require one to have access to the evolutionary process transforming *G*_1 _into *G*_2_, and keep the trace of the correspondence between telomeric and fictional elements. We can nonetheless build such a correspondence, which is used also for example in [15]. The union of observed adjacencies of *G*_1 _and *G*_2 _is composed of a set of disjoints cycles and paths. Let *P *be a path. If *P *has an even number of edges, then it starts with an adjacency of *G*_1 _and ends with an adjacency of *G*_2_. Take a telomeric vertex *t *and join it to the two extremities, creating a telomeric adjacency in *G*_1 _and a telomeric adjacency in *G*_2_. So *P *is turned into a cycle. Now if *P *has an odd number of edges, suppose it starts and ends with adjacencies from *G*_1 _(the other case is symmetric). Take two fictional elements *t*_1 _and *t*_2_, join each of them to one different extremity of *P *, with telomeric adjacencies from *G*_2_. Then join *t*_1 _and *t*_2 _with a fictional adjacency from *G*_1_. *P *is again turned into a cycle. If there remains unmatched telomeres or fictional elements, make trivial cycles of two parallel edges from *G*_1 _and *G*_2 _out of them. The obtained set of disjoint cycles is called the *observed breakpoint graph*, and it is always possible to construct it from two sets of observed adjacencies for two genomes on the same set of genes.

The observed breakpoint graph has a maximum number of cycles, given its observed adjacencies. As it shares the observed adjacencies with the real breakpoint graph, the number of cycles in the observed breakpoint graph is never lower than in the real breakpoint graph. The difference between the two is bounded by the number of telomeric and fictional vertices, because in the extreme case, there is one cycle per telomeric or fictional vertex in the observed breakpoint graph, and one cycle containing all vertices in the real breakpoint graph. By the assumption that *f *and 2*g *− 2*a *are both *o*(*n*), we can also assume that the difference between the two numbers of cycles is bounded by *o*(*n*), so that the number of cycles in the real breakpoint graph can be estimated with a bounded error.

### Cycles of permutations and breakpoint graphs

An analogy can be stated by using the identity permutation *Id *as a starting point, as the genome *G *as the starting point, and applying successive transpositions to *Id*, as DCJs are applied to *G *(see Figure 1(A) and 1(C).

• adjacencies in *G *(observed or not) are identified with elements in the identity permutation *Id*;

• cycles of the breakpoint graph of *G *and *G_k _*are identified with cycles of the permutation *P_k _*obtained from *Id *by a series of *k *transpositions;

• a DCJ can increase, decrease or leave unchanged the number of cycles, while a transposition can increase or decrease the number of cycles.

Transpositions in the symmetric group are a case of coagulation-fragmentation processes, since at each step either a fission splits a cycle into two, or a fusion joins two cycles into one. DCJ adds a third possibility, because the number of cycles may stay unchanged. Eriksen and Hultman [9] proposed an exact formula for the expected number of cycles in a permutation obtained from the identity permutation of size *n *by a series of *k *random transpositions:

(5) $$E\left(Cy_{k}\right)=n-\overset{n}{\underset{i=1}{\sum }}\frac{1}{i}+\overset{n-1}{\underset{p=1}{\sum }}{\overset{\text{min}\left\{p,n-p\right\}}{\underset{q=1}{\sum }}a_{pq}\left(\frac{\left(\begin{array}{c} p\\ 2 \end{array}\right)+\left(\begin{array}{c} q-1\\ 2 \end{array}\right)-\left(\begin{array}{c} n-p-q+2\\ 2 \end{array}\right)}{\left(\begin{array}{c} n\\ 2 \end{array}\right)}\right)}^{k},$$

where

$$a_{pq}={\left(-1\right)}^{n-p-q+1}\frac{{\left(p-q+1\right)}^{2}}{{\left(n-q+1\right)}^{2}\left(n-p\right)}\left(\begin{array}{c} n-p-1\\ q-1 \end{array}\right)\left(\begin{array}{c} n\\ p \end{array}\right).$$

Simulations showed that it was a rather precise way to estimate the number of rearrangements but no formal link was established. We prove that it approximates the expected number of cycles in the breakpoint graph of genomes *G *and *G_k _*, where *G_k _*is obtained from *G *by *k *random DCJs.

**Theorem 2 ***Let BCy_k _be the number of cycles of the breakpoint graph between a genome G with n genes and a genome G_k _evolved from G by k random DCJs, and Cy_k _be the number of cycles of a permutation evolved by k transpositions from the identity permutation with n elements. Then*

$$E\left(BCy_{k}\right)=E\left(Cy_{k}\right)+o\left(n\right).$$

This remains valid for the real or observed breakpoint graph.

*Proof *Let *G *be a genome with *n *adjacencies and *Id *the identity with *n *elements. We apply a DCJ process on *G*, and it will imply a transposition process on *Id*. Elements of *Id *can be mapped to adjacencies of *G*, as cycles of the breakpoint graph of *G *and *G*_0 _can be mapped to cycles of *Id*. At any step of the process, we will keep this mapping between elements of *P_k _*and adjacencies in *G_k _*. The mapping between the types of cycles will be less strict because of the difference between the two processes.

When a DCJ cuts adjacencies *a *and *b *on the current genome, we also apply the transposition *ab *to the current permutation.

If *a *and *b *are in two different cycles of the breakpoint graph, then the two cycles are necessarily joined by a fusion into one, just as a transposition on two different cycles fusions them into one. In that case the processes are identical from the point of view of cycles. The two new adjacencies arising from the DCJ are mapped to the elements *a *and *b *in the resulting permutation, in an arbitrary way.

If *a *and *b *are in the same cycle of the breakpoint graph then, with probability 0.5, the cycle is splitted into two, and with probability 0.5 the cycle is unchanged (only the order of the elements are changed). In the permutation, the cycle is necessarily splitted by a fission into two new cycles. In the case of a fission of the cycle in the breakpoint graph, the two new adjacencies are mapped to elements *a *and *b *of the permutation, in order to respect the cycles: if the new adjacency goes into a cycle of the breakpoint graph with some adjacencies that are mapped to the elements going into a cycle of the permutation with *a*, then map it to *a*. In the case the breakpoint graph is unchanged from the point of view of the cycle distribution, map the new adjacencies to *a *and *b *arbitrarily.

The permutation clearly follows a process of evolution by random transpositions. Moreover, the correspondence between the processes ensures that the number of cycles of the breakpoint graph is always lower than the number of cycles in the permutation. Indeed, every time the number of cycles decreases, it decreases in both processes. And every time the number of cycles increases in the breakpoint graph, it also increases in the permutation. So we have

$$E\left(BCy_{k}\right)\leq E\left(Cy_{k}\right).$$

The difference between the two will be bounded by the number of times a DCJ occurs on *a *and *b *in the same cycle, and the number of cycles in the breakpoint graph remains unchanged. The probability that a cycle of fixed size $s<\sqrt{n}$ is created from a fission in the permutation but not in the breakpoint graph is less than 1/*n*. So the probability to create a cycle of any size at most $\sqrt{n\;}$ is less than 1/$\sqrt{n\;}$. The expected number of such events is thus less than $k/\sqrt{n}$, and as the number of cycles of size at least $\sqrt{n\;}$ cannot itself be more than $\sqrt{n\;}$, we have, for $k\geq \sqrt{n}$,

$$E(Cy_{k})\leq E(BCy_{k})+O(k/\sqrt{n}).$$

As we can suppose that *k *is always under *O*(*n *log *n*) because after this the signal is saturated, it proves the result, provided the cycles of the breakpoint graphs are known. However we saw in the introduction that the real breakpoint graph is not always known. If they are unknown they can be approximated within a *o*(*n*) factor. So we get the result even if we do not have access to the common telomeric and fictional structure of the genomes.

## A link with random graphs

Berestycki and Durrett [10] proposed, as an analogy with signed inversions evolving by reversals, to use the evolution of random graphs [16]. The analogy can be stated with DCJ, by using a random graph model starting from an empty graph *Gr*, and adding random edges among the $\left(\begin{array}{c} n\\ 2 \end{array}\right)$ possible ones at each step. Note that we allow parallel edges, so the model is not exactly Erdős and Rényi's, but most parameters evolve in the same way. The relation between genomes and graphs is the following, depicted in Figure 1(A) and 1(B):

• adjacencies in *G *are identified with vertices in the empty graph *Gr*;

• cycles of the breakpoint graph of *G *and *G_k _*are identified with connected components of *Gr_k _*, obtained from *Gr *by adding a series of *k *edges;

• a DCJ can increase, decrease or leave unchanged the number of cycles, while adding an edge can decrease or leave unchanged the number of cycles.

We noticed that DCJ was a sort of "coagulation-fragmentation-nothing" process, compared to the "coagulation-fragmentation" behavior of transpositions in the symmetric group. Here random graphs can be considered as "coagulation-nothing" processes, since an edge can fusion two connected components or change nothing to the distribution of components if it falls inside one. Berestycki and Durrett [10] proved a relation between the process of transpositions in permutations and random graphs:

**Theorem 3 **(Berestycki and Durrett (Theorem 3) [10]) *Let Co_k _be the number of components of a graph Gr evolved from the empty graph with n vertices by adding k random edges, and Cy_k _be the number of cycles of a permutation evolved by k transpositions from the identity permutation with n elements. Then*

$$E\left(Cy_{k}\right)=E\left(Co_{k}\right)+O\left(\sqrt{n}\right).$$

There does not seem to exist a good computable general formula for the number of components of a graph after the addition of *k *edges. Berestycki and Durrett [10] use the formula for the number of trees:

(6) $$\overset{\infty }{\underset{i=1}{\sum }}\frac{n}{2k}\frac{i^{i-2}}{i!}{\left(\frac{2k}{n}e^{-\frac{2k}{n}}\right)}^{i},$$

which is a provably good approximation of it, and can be considered computable if we neglect the high terms of the sum. But they did not prove a relationship of the estimator with a rearrangement model, though their study was motivated by inversions. A direct corollary of Theorems 2 and 3 is

**Corollary ***Let BCy_k _be the number of cycles of the breakpoint graph between a genome G with n genes and a genome G_k _evolved from G by k random DCJs, and Co_k _be the number of components of a graph Gr evolved from the empty graph with n vertices by adding k random edges. Then*

$$E\left(B_{k}\right)=E\left(Cy_{k}\right)+o\left(n\right).$$

## Empirical comparisons

We tested all estimators on the same set of simulated genomes, evolving by DCJ according to our model. We started with *n_G _*= 980 genes, and matched them randomly to make a starting genome *G*, so that 40 vertices among the gene extremities remain unmatched. We added 10 fictional elements. Then we performed a random DCJ at each step *k *during 4000 steps, then obtaining 4000 genomes *G_k _*.

At each step we computed:

• *DCJ *, the DCJ distance, which is the minimum number of DCJs between *G *and *G_k _*;

• $\tilde{DCJ}$, the estimator presented here with a closed and exact formula;

• *EH *is the approximation derived from the link with transpositions and symmetric groups, based on the work of Eriksen and Hultman [9];

• *BD *is the approximation inspired by the relation between evolution of random graphs and genome rearrangements, pointed out by Berestycki and Durrett [10];

• *LM *is the heuristic described by Lin and Moret [2].

In terms of running time, *EH*, *BD *and *LM *all require the precomputation of expected values for 4000 DCJs, and at each step the search for the value of *k *which minimizes the observed and expected parameter. For *LM *, which has no closed formula but does have a recursive one, this precomputation and this mode of numerical inversion is necessary, while for *EH *and *BD*, as they have a closed formula, a smarter numerical inversion could be imagined. The running time of *EH *was quite high, since it required very high precision numbers. The running time of *BD *depends on where we decide to cut the infinite sum of Equation 6. For $\tilde{DCJ}$, the computations are nearly instantaneous, as there is a closed, analytically invertible formula that does not require any sum.

**Figure 4:**
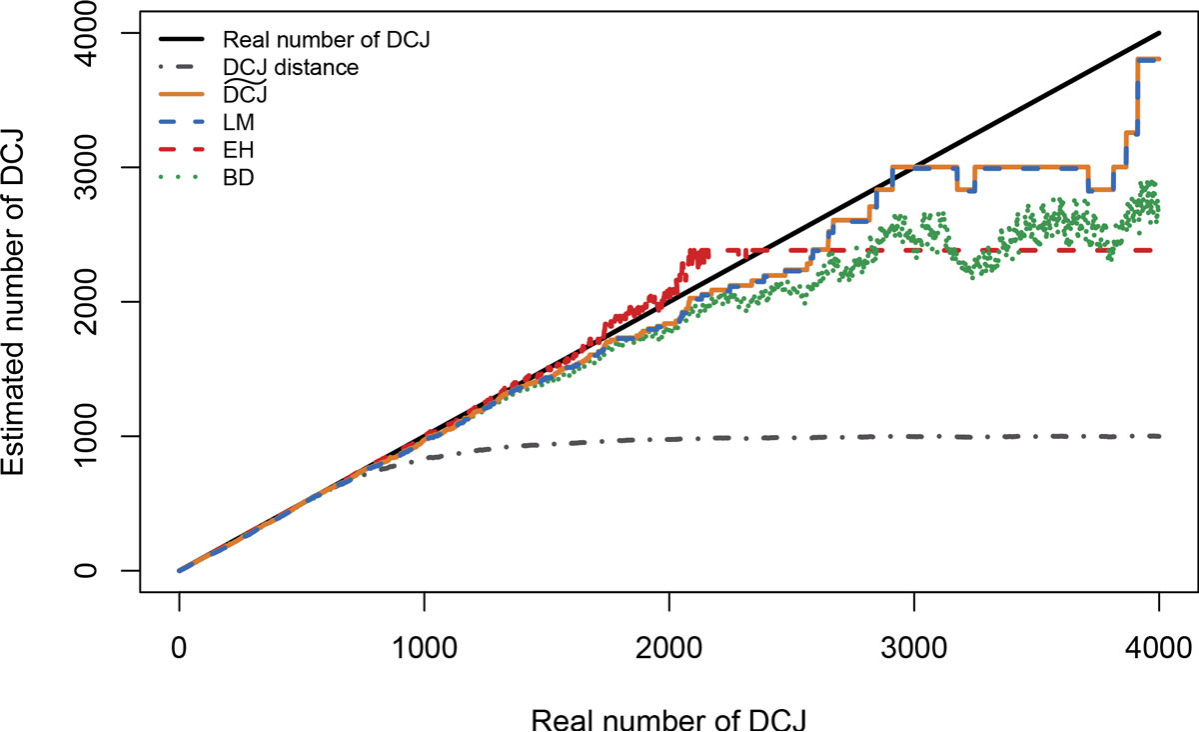
**Comparisons of some DCJ estimators on a simulation from the DCJ model on *a *= 980 observed adjacencies and *n *= 1020 adjacencies in total**. LM is the estimator from [2], EH from [9], BD from [10], and $\tilde{DCJ}$ is our formula. The approximate LM estimator is indistinguishable from our exact one, except by its higher running time. Other estimators give slightly different values but are comparable in quality.

Results are given in Figure 4. Such graphs are present in all publications related to a single estimator but were never compared. They tend to have approximately the same behavior, and from a single run no striking difference can be assessed. They estimate a number close to the real one approximately twice as long as parsimony (*DCJ *) does, and then the estimate starts to diverge away from the diagonal, though still much better than parsimony. The quality of *LM *and $\tilde{DCJ}$ are indistinguishable, though one is exact and the other heuristic. So the main empirical advantage of $\tilde{DCJ}$ seems to be the running time, in addition to the theoretical value of having a closed formula. *LM *was computed in 2.628 seconds, whereas $\tilde{DCJ}$ took only 0.015 seconds.

## Discussion/Conclusion

Statistical estimators of rearrangement distances are very diverse, using the similarity to various random processes as coagulation-fragmentation, sequence evolution, random graphs, transpositions in the symmetric group, being more or less approximate, tractable in practice, and using different parameters from the genome comparisons, *e.g*. number of breakpoints or number of cycles in the breakpoint graph.

They all suppose a model where events are more or less equiprobable, even if, as we saw, there can be slightly different interpretations of this in the case of DCJ. But this makes them comparable in a single framework, both theoretically and empirically, as we tried to do in this contribution.

An interesting difference are estimations based on number of breakpoints or number of cycles in the breakpoint graph. The distribution of cycles in the breakpoint graph contains the information of the breakpoints because breakpoints are all observed adjacencies in non trivial cycles, that is, not in cycles formed by parallel edges. So the distribution of cycles should contain more information than the breakpoints. But in practice they seem to carry the same information. In particular the saturation of the information happens at the same time, or at least same order. It was remarked by Caprara and Lancia [7] that a permutation is randomised after *O*(*n *log *n*) inversions. It means that after this number of rearrangements, there will be no signal in breakpoints or in the breakpoint graph to retrieve any evolutionary distance. This bound is also the time after which random graphs starting from empty graphs get connected almost surely [16]. The analogy between random graphs and genomes translates this result into: after *O*(*n *log *n*) DCJs, it is expected that no adjacency remains unbroken, so the number of breakpoints becomes meaningless for a distance computation. So statistically breakpoints seem to contain as much information as cycles of the breakpoint graph, and as they are often easier to compute, from this statistical point of view they are more promising for phylogeny.

We remark as a curiosity that mathematical and computational difficulties are not necessarily correlated for combinatorial and statistical problems. For example, sorting unsigned permutations by a minimum number of inversions is NP-hard, while estimating the number of breakpoints after a fixed number of inversions has a nice solution [7]. Sorting signed permutations by a minimum number of inversions is polynomial, while no closed exact formula exists so far for the statistical problem (but has an approximate nice solution [17]). Only for DCJ do we have simple solutions in both cases. Sorting a permutation by a minimum number of transpositions is a known difficult combinatorial problem, and statistical solutions are not known but do not seem out of reach [6].

## Competing interests

The authors declare that they have no competing interests.

## Authors' contributions

PB, LG and ET stated the theorems and constructed the proofs. PB performed the simulations. PB and ET wrote the article.
